# Supplementary material for: Linking Pharmacogenomic Information on Drug Safety and Efficacy with Ethnic Minority Populations
Source: Pharmaceutics. 2020 Oct 25;12(11):1021. doi: 10.3390/pharmaceutics12111021 (PMC7693750; doi:10.3390/pharmaceutics12111021)
Supplement: Supplementary file 1 [file pharmaceutics-12-01021-s001.zip › pharmaceutics-976333-supplementary figure and table.docx]

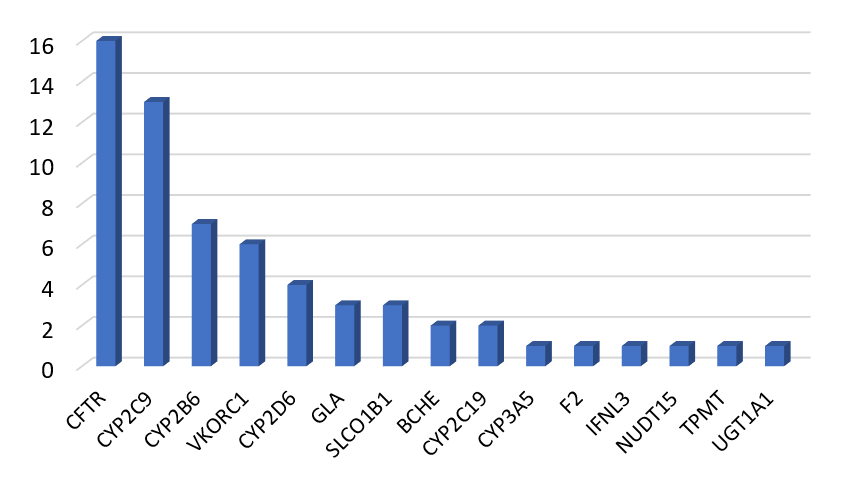


**Figure 1.** The number of SNPs in each biomarker that were associated with drugs in TPGxBMDL.


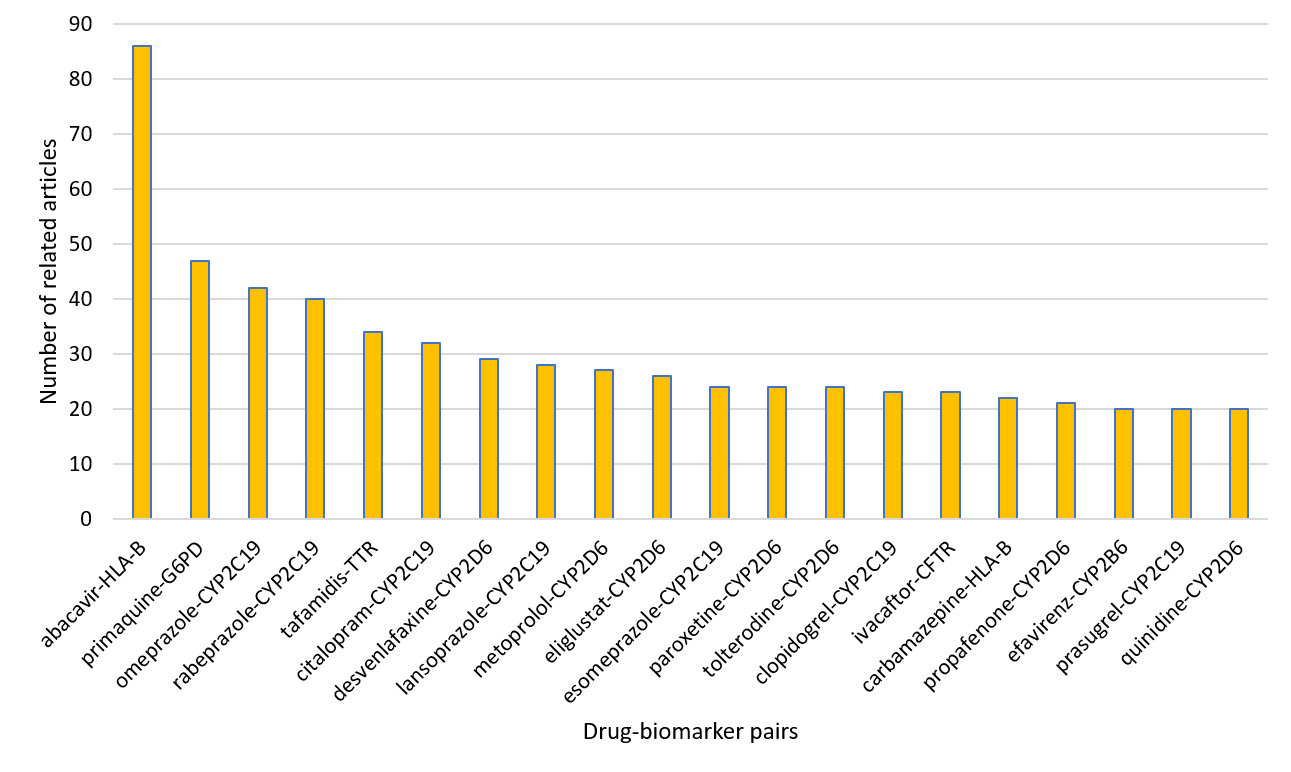


**Figure 2.** The number of PubMed articles that mentioned the drug-biomarker pairs and ethnic group key words in the abstracts. In total of 120 drug-biomarker pairs covered by 1329 articles were obtained and only the pairs with more than 20 articles were shown in this figure.

**Table 1.** Ethnic groups and subject information included the ALFA project (version 20200227123210).

| Ethnic Group | ID | Description | Subjects |
| --- | --- | --- | --- |
| African | SAMN10492703 | All Africans, AFO and AFA Individuals | 3,669 |
| African Others | SAMN10492696 | Individuals with African ancestry | 114 |
| Asian | SAMN10492704 | Asian Individuals excluding South Asian | 237 |
| East Asian | SAMN10492697 | East Asian (95%) | 153 |
| South Asian | SAMN10492702 | South Asian | 2,459 |
| Other Asian | SAMN10492701 | Asian individuals excluding South or East Asian | 84 |
| African American | SAMN10492698 | African American | 3,555 |
| Latin American 1 | SAMN10492699 | Latin American individuals with Afro-Caribbean ancestry | 354 |
| Latin American 2 | SAMN10492700 | Latin American individuals with mostly European and Native American Ancestry | 3,801 |
| European | SAMN10492695 | European | 82,475 |
| Other | SAMN11605645 | The self-reported population is inconsistent with the GRAF-assigned population | 5,499 |
| Total | SAMN10492705 | Total (~global) across all populations | 98,494 |
